# Low Velocity Drop-Weight Impact of Flax–Glass Hybrid Composites for Application in Automotive Components: Numerical Modelling and Experimental Analysis

**DOI:** 10.3390/ma18204740

**Published:** 2025-10-16

**Authors:** Tegginamath Akshat, Michal Petru, Rajesh Kumar Mishra

**Affiliations:** 1 Department of Machine Parts and Mechanism, Faculty of Mechanical Engineering, Technical University of Liberec, Studentská 1402/2, 461 17 Liberec, Czech Republic; akshattm93@gmail.com (T.A.); michal.petru@tul.cz (M.P.); 2 Department of Material Science and Manufacturing Technology, Faculty of Engineering, Czech University of Life Sciences Prague, Kamycka 129, Suchdol, 165 00 Prague, Czech Republic

**Keywords:** drop-weight impact, EPS foam, flax, glass, hybrid composite, LS-DYNA

## Abstract

This study focuses on the behavior of hybrid polymer composites made from glass fiber and natural fiber-based flax fabric when subjected to low velocity drop-weight impacts. With the rise in the utilization of composites in structural components in various industries like the marine, aerospace and automotive industries, it is of paramount importance to study the effects of low velocity drop-weight impacts and their damage assessment on the composites. Low velocity drop-weight impacts can occur due to a tool falling on a composite part or due to an impact with a small object. The experimental tests were carried out according to ASTM standards with a drop-weight impact testing machine. Simulations were done to replicate the tests using explicit finite element software LS-DYNA. The experimental tests were carried out on samples of thickness ~2.5 mm and the energy at impact was 50 J. Upon comparing the experimental results, it was seen that an error percentage in the deformation varied between a minimum of 3.32% and a maximum of 8.93%, and the maximum force at impact varied between a minimum of 0.06% and a maximum of 17.14%. The variations between the experimental and simulated values can be attributed to the presence of voids or other defects that would have inadvertently crept in while making the composite. Additionally, composite laminates lined with a layer of EPS (expanded polystyrene) foam were tested and compared with composite laminates which were not lined with the foam. An improvement in the performance of the composite laminates lined with the EPS foam was observed.

## 1. Introduction

Traditionally, internal components and frames of automobiles have been made from metals. The usage of metals for the internal components and frames tends to increase the overall weight of an automobile. In order to reduce the overall weight of automobiles, fiber-reinforced composites are being developed as a replacement. The main characteristic of these fiber-reinforced composites is that they are light weight and also offer a high degree of stiffness. Thus, it can be observed that the extensive use of these composite materials in the transportation industry can help achieve a high degree of mechanical performance along with improving the efficiency of vehicles due to the reduction in weight they offer [1]. Over the last decade, an increase in the interest shown towards improving the environmental impact of the materials used in making these composites has led to the development of sustainable and eco-friendly composite materials which incorporate natural fibers as reinforcing materials [2,3,4], and composite materials in general offer an uncanny advantage where the properties of the materials used can be modified in order to meet the various requirements of the applications [1].

The ultimate microstructure of the composite, which is a result of compaction, partially dictates the mechanical properties of a composite [5,6]; along with this, the mechanical properties of the yarn utilized, which are dependent on the cross section of the yarn, are also responsible for the mechanical properties of the composite material [1]. The mechanical properties of fiber-reinforced composites are dependent on the yarn fiber volume ratio, which has a range of 0.76 to 0.9; for the case of an ideal structure with unidirectional hexagonal packing, the yarn fiber volume ratio is found to be 0.98 [7,8]. The macroscopic behavior of the heterogenous fiber-reinforced composite is dictated by the geometrical arrangement of the phases and the properties of the interface [9]. The fiber volume fraction is an important parameter which has a greater influence on the mechanical properties of the fiber-reinforced composites, as seen by the studies conducted by some researchers on the fiber packing density of idealized yarn constructions and the distribution of fibers in the cross section [8,10,11,12]. Hybrid composites have been front and center on the world stage in recent years due to their versatility and the range of applications for which they can be utilized, as well as the fact that the properties of the composites can be easily modified to satisfy the requirements of potential applications. The rise in environmental awareness, as mentioned earlier, has led to the development of sustainable and eco-friendly composites. These composites are made from a combination of high-performance fibers like carbon, Kevlar, glass, etc. with natural fibers like sisal, jute, hemp, flax, etc. [13]. It is evidently clear that the mechanical performance of the natural fibers when compared with the mechanical performance of the high-performance fibers is lower; however, we can utilize the high-performance fibers as the outer layers of the composite while utilizing the natural fibers as the inner layers of the composite, thus creating a hybrid composite which is sustainable and ecofriendly in nature with multiple reinforcements [14]. These hybrid composites can be utilized in the marine, automotive and aviation sectors, as well as for with manufacturing blades for turbines and producing numerous household items.

The frames and components which are made from traditional materials can be replaced by components and frames made from high performance materials like basalt, carbon, Kevlar, etc. These components or frames can be converted into composites using resins. The frames and components made from these materials are ultra-light weight in nature and are mechanically sturdy; to predict the performance of these frames and components, advanced computational tools can be used [15,16,17,18,19,20,21,22]. Zero toxicity, nonabrasive nature, biodegradability, lower costs and ease of manufacturing are some of the advantages that the natural fibers offer over the synthetic fibers [23,24,25,26,27,28]. Usually, natural fiber-based reinforced composites are made from plant fibers such as hemp, jute, sisal, flax, banana, etc. [29,30]. Investigations into the physical and mechanical properties of natural fiber-based composites made from sisal, hemp, jute, coir, arrowroot, kenaf, pinecone and bamboo have been conducted in detail by various authors [31,32,33,34,35,36]. The thermal and mechanical properties of the hybrid composites can be increased significantly when synthetic fibers or high-performance fibers are added along with the natural fibers. The tensile properties [37,38,39,40], flexural [41], impact strength [42,43,44,45], damping [46] and interlaminar shear strength [41] of the hybrid composites have been investigated to determine the effect of hybridization.

The eco-friendliness, ease of handling, biodegradability, carbon neutral nature, cost effectiveness, higher specific strength and excellent mechanical properties are some of the properties exhibited by flax fibers, and this has led to the extensive research that has been conducted on flax-based composites. The composites made from flax and other natural fiber-based composites have a wide range of applications in the automotive sector, the biomedical sector, the production of turbine blades, the marine industry, the aerospace industry, printed circuit boards and the construction industry [47,48,49,50,51,52,53,54,55,56,57,58,59,60,61,62,63].

The mechanical properties of composites due to hybridization were studied by several researchers [46]. Vacuum assisted transfer molding process was utilized to produce the composite samples. Flax, carbon and glass were used as reinforcing fibers in the composites and the main reason for the integration of flax was to make the composite environmentally friendly. Tensile strength, impact strength, flexural strength, interlaminar shear strength and dampening properties were studied in this study. The results obtained from the impact tests revealed that the hybrid composites which had reinforcing fibers of flax and glass had the highest impact strength when compared to the other composite samples tested.

A study conducted by researchers [64] dealt with the effects of stacking sequence on the mechanical properties like tensile strength, flexural strength and impact strength. The hybrid composites were prepared with kenaf fiber fabrics and glass fiber fabrics. The results obtained from the impact tests revealed that the hybrid composites had a superior performance where there was an improvement in the impact strength in the edgewise orientation of 8.6% and an improvement of 51.9% was shown in the flatwise orientation.

The tensile, flexural and impact strengths of flax and hemp-based hybrid composites when exposed to a cryogenic treatment were evaluated and compared with composite samples which were exposed to a cryogenic treatment [65]. The composites were prepared using the hand lay-up method and the fibers used in the preparation of the composites were treated with NaOH solution to increase their interfacial adhesion. The results of the experiments conducted showed that the impact strength of the composite samples decreased with the increase in the immersion time in the liquid nitrogen which was used to administer the cryogenic treatment.

Researchers conducted experimental investigations for the usage of hemp fiber-based hybrid composites for automotive applications [66]. The hemp fibers were integrated with glass fiber reinforced composites. Both treated and untreated fibers were integrated with the glass fiber reinforced composite. The treated fibers were treated with NaOH and the mechanical properties like tensile strength, impact strength and hardness were tested. The samples were fabricated using the hand lay-up method. The results obtained from the impact tests show that the hybrid composites have good impact strength when compared to the glass epoxy composites.

The effect of stacking sequence on the mechanical and thermal properties of flax, Kevlar, carbon and carbon–Kevlar hybrid epoxy composites were studied [67]. In the study, tensile strength, flexural strength, interlaminar shear strength, impact strength, thermal behaviors, water absorption and contact angle were evaluated. The composites produced had four layers and were produced using the hand lay-up method. The impact tests revealed that the performance of the hybrid composites were better than the other samples showing an impact strength of 13.86 MPa. The structural integrity and the strength of the composite materials can be significantly diminished due to the damage caused by drop-weight impacts, and visual inspection of these damages may not be enough to detect them. This is due to the fact that the damages induced by these impacts occur internally and may increase when minor delamination of the composite occurs. Thus, it is of paramount importance to study the behavior of these laminated composites upon impact [68]. It has been seen that the composites made from woven fabrics exhibit superior resistance to impact damage owing to the fact that there are interlacing fiber tows present in two directions, and along with this they are easy to manufacture, exhibit high toughness and have better damage tolerance [69].

With low energy impacts on rectangular woven E-glass polyester composites of varying thickness, researchers have shown that complex, multiple damages occur [70]. Several researchers also conducted a study using finite element analysis in ABAQUS version 2025X FD01 for low velocity impacts on thin composite laminates [71]. In a study, the energy-based damage mechanics were evaluated using DYNA3D version R14.1.0 to study the effect of impact on carbon fabric composite laminates [72]. To obtain a clear picture of the damage caused due to impacts, it is also necessary to compare results obtained after simulation with the experimental results. A study was conducted where energy absorption and impact damage resistance for unidirectional, 2D plane woven and single-ply 3D orthogonal fabric laminates were analyzed. The results of the analysis indicated that the single-ply 3D orthogonal fabric laminate had a greater level of impact damage resistance and higher energy absorption capability when compared with the other two type of fabric laminates [73].

Traditionally, the models used for the analysis of impact damage have relied heavily on experimental data or analytical calculations. It has been observed that the analytical results are overly simple and unreliable, and it tends to become very expensive to conduct tests for each and every design, with the testing being complex and time consuming. Thus, the virtual testing of the desired sample using numerical simulation is catching on for engineering development. As a result, various geometries, laminate configurations and loading conditions can easily simulated and their effects can be analyzed.

Based on the research gap evident from the available literature, the impact performance of laminated hybrid composites was the focus of this investigation. In this study, the behavior of hybrid polymer composites made from glass fiber and natural fiber-based flax fabric, when subjected to low velocity drop-weight impacts. The effect of the low velocity impact might not be apparent as the energy would be transmitted through the material, leading to the formation of minute cracks or deformations which over time could grow in size and affect the structural integrity of the part, and ultimately to a catastrophic failure of the component. In this work, tests have been conducted to determine the maximum force which the composite can withstand before experiencing a failure. The experimental tests were carried out according to ASTM standards with a drop-weight impact testing machine. Simulations were done to replicate the same using explicit finite element software LS-DYNA version R14.1.0. The results obtained from the experimental tests were compared with the simulated results in order to determine the error of prediction. Additionally, composite laminates lined with a layer of EPS (expanded polystyrene) foam were tested and compared with composite laminates which were not lined with the foam. An improvement in the performance of the composite laminates lined with the EPS foam was expected.

## 2. Materials and Methods

### 2.1. Materials

#### Sample Preparation

The materials used to make the composite laminates for the low velocity drop-weight impact tests had the properties as shown in Table 1.

**Figure 1:**
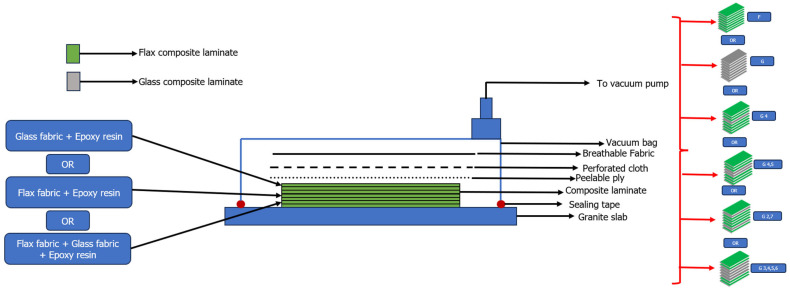
Schematic showing preparation of composite laminates. Adapted from [2].

The composite laminates were prepared via the hand lay-up process, followed by the vacuum bagging process. The schematic representing the process of preparation of the samples is given in Figure 1.

The composite laminates were prepared using the vacuum bagging process; the samples which were produced had 8 layers of fabrics, each with a thickness of 2.5 ± 0.1 mm and a fiber volume fraction of 0.45. A total of 18 samples for the purpose of low velocity drop-weight impact testing were cut from the composite laminates. The samples were pure glass fabric, pure flax fabric and composites which were a combination of different layers of glass and flax fabrics.

LH 288 epoxy resin, which is a two-component structural epoxy resin, was used with a H 282 hardener from Havel Composites CZ s. r. o., Prague, Czech Republic. This matrix is characterized by a low viscosity of 500–900 mPa·s at 25 °C. The density was 1100–1200 kg/m^3^ at 25 °C. The volume fraction of matrix used for impregnation was 0.55.

Table 2 shows the samples which were prepared for testing:

### 2.2. Experimental Testing

#### Low Velocity Drop-Weight Impact Testing

**Figure 2:**
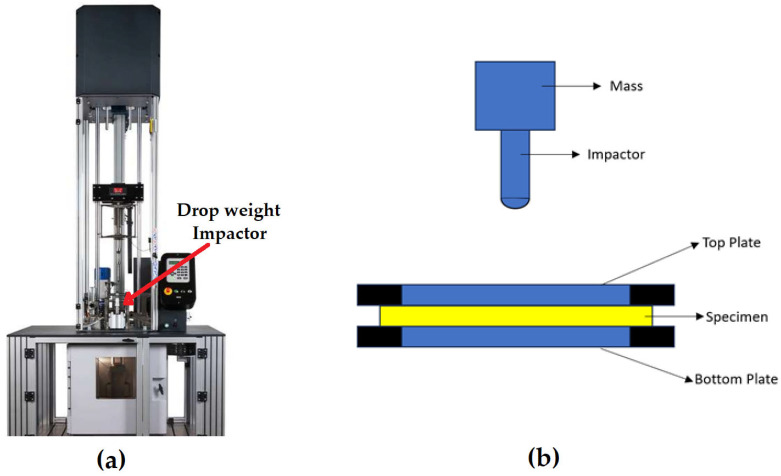
(**a**) Low velocity impact tester; (**b**) schematic of drop-weight impact tester.

The picture and schematic of the low velocity drop-weight impact tester used for testing the composite laminates are given below in Figure 2. The machine used for testing had a sensor for velocity, load cell, an accelerometer, a weight assembly and an impactor. The data which was generated was stored and converted to energy absorbed, contact force and displacement. The tests were conducted according to ASTM D 7136 [74]; this is the method which is commonly used to determine damage resistance of a polymer matrix composite when they are impacted by a drop-wight [75]. A hemispherical impactor of diameter 8 mm was attached to the end of the load cell. The impactor used here was assumed to be perfectly rigid. For the current study, composite laminates with dimensions 100 mm × 100 mm × 2.5 mm were used. When being tested, the laminates were clamped between the top and bottom plates, as shown in schematic in Figure 2. The point of impact was located at the center of the composite laminate. All the samples were impacted with a constant velocity and from a constant height.

The energy at impact of the impactor can be calculated using formula, E = mgh, where: E—Energy (J), m—mass (kg), g—acceleration due to gravity (9.81 m/s^2^) and h—height (m).

### 2.3. Methods

#### 2.3.1. Modelling Software

Using SpaceClaim version 2024.R2 in ANSYS 2024, a 3D model of the composite laminate with dimensions 100 mm × 100 mm × 2.5 mm was made.

To simulate the model in ANSYS 2024 and predict the performance, the yarn/tow model for a single element was considered, as this would lead to reduced simulation times compared to when multiple filaments models would be used.

#### 2.3.2. Modelling Methodology

**Figure 3:**
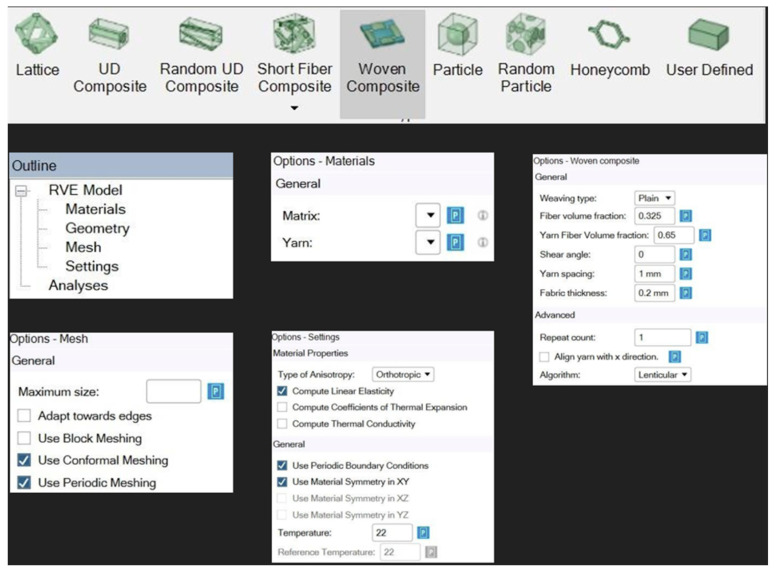
Parameters chosen to create a woven fabric. Adapted from [2].

**Figure 4:**
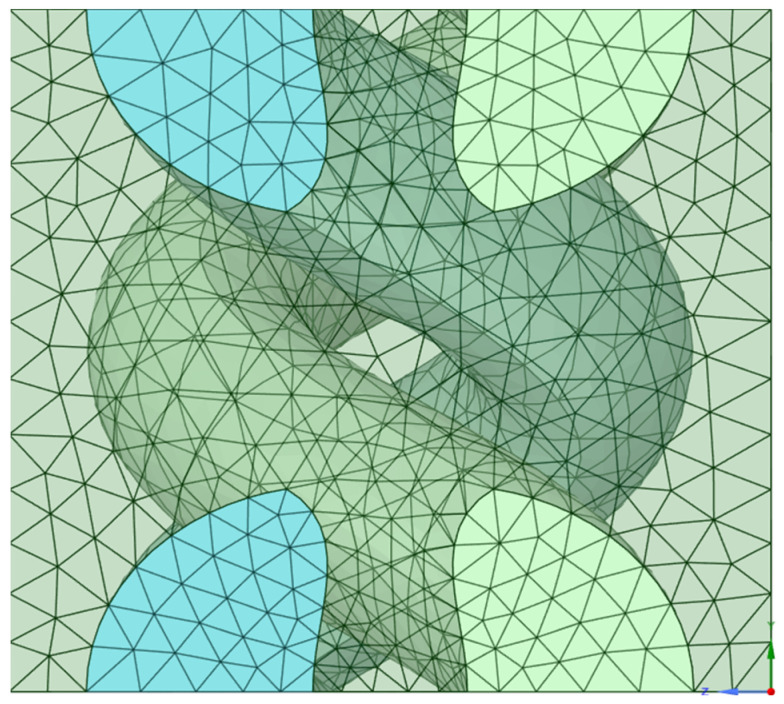
Basic repeating unit of woven fabric in SpaceClaim of Material Designer Module. Adapted from [2].

In the material designer module of ANSYS, a model of a woven fabric was created which later used in the creation of the various layers which were stacked and used for the modelling and testing of the sample in ANSYS. To create the fabric in the designer module of ANSYS, woven fabric option was chosen and the relevant details pertaining to the fabric like yarn spacing, yarn volume, shear angle, yarn volume fraction and thickness of the fabric were entered. After this, the type of matrix and the type of yarn to be used were also entered. The various parameters that were chosen to make the woven fabric in the Material Designer module of ANSYS are shown in Figure 3 and Figure 4, representing a repeating unit with a meshing pattern for the woven fabric.

Figure 4 represents the woven fabric created in the designer module, where the properties of the fabric, properties of the fiber used, and the resin used were fed into the designer module and a unit cell was created to demonstrate the woven fabric. The figure also shows the mesh generation for the unit cell.

**Figure 5:**
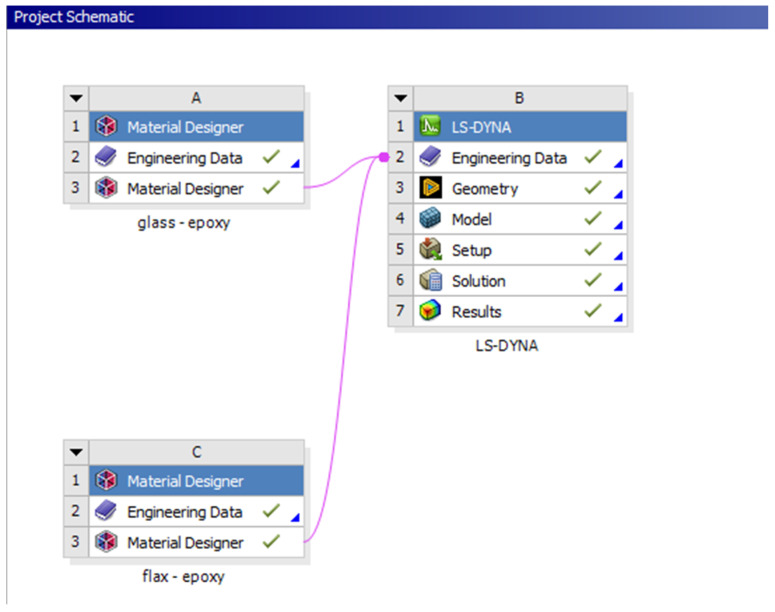
Project schematic used.

The data of the fabrics created were then fed into the Engineering data of the LS-DYNA module of ANSYS, as shown in the project schematic in Figure 5.

In order to create a composite with numerous layers of woven fabric in ANSYS, the woven fabrics must be created first. The option of woven composite is selected and details of the woven fabric (weave type, yarn volume fraction, shear angle, yarn spacing and fabric thickness) are entered, along with selection of the yarn type and the matrix. After which, the meshing parameters and other material properties like the orthotropic nature of the material is selected.

All samples were created in ANSYS using the following steps:

Step 1: Creating the composite fabric: To create a composite fabric in material designer the following data has to be fed; weave type, yarn spacing, fiber volume fraction, thickness. The data which is provided defines the fabric and helps in creating the fabric virtually, and a set of data is generated in the Material Designer along with the generation of the fabric.

Step 2: The data that is generated in the Material Designer is transferred to the Engineering Data module in Workbench.

Step 3: The data from Engineering Data module is then transferred to the Engineering data section of the ACP (Pre) module.

Step 4: Using CAD (SolidWorks) software, version 2025 a geometry of the sample is created, which is then imported to Design Modeler in ANSYS.

**Figure 6:**
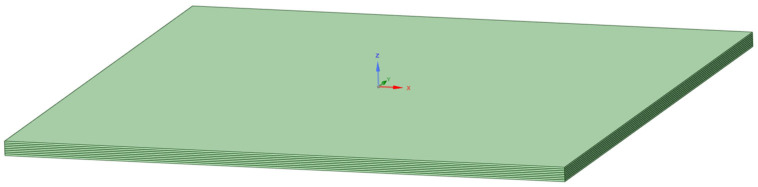
Geometry showing 8 layers.

Step 5: The fabrics in the setup module are added and the fabric properties are defined for all 8 fabric layers. The geometry of the sample with 8 layers was created in Space-Claim, as shown in Figure 6, where the thickness of each layer was defined.

Here the thickness is defined as 0.3125 mm as the sample has 8 layers with a total thickness of 2.5 mm, and therefore the thickness of one layer of fabric is given by (2.5/8) which is 0.3125 mm.

Step 6: The elements which are to be taken into consideration for testing are selected.

Step 7: Rosette and Oriented Selection Set are defined, and modelling ply is created with the appropriate properties.

Step 8: After creating the plies with the appropriate properties, the data is transferred to the static structural module.

Step 9: In the static structural, the simulation was run to determine the impact strength (peak load) values for the given sample.

Step 10: To simulate the rupture of the samples, the data generated is transferred to the explicit dynamic module (LS-DYNA).

**Figure 7:**
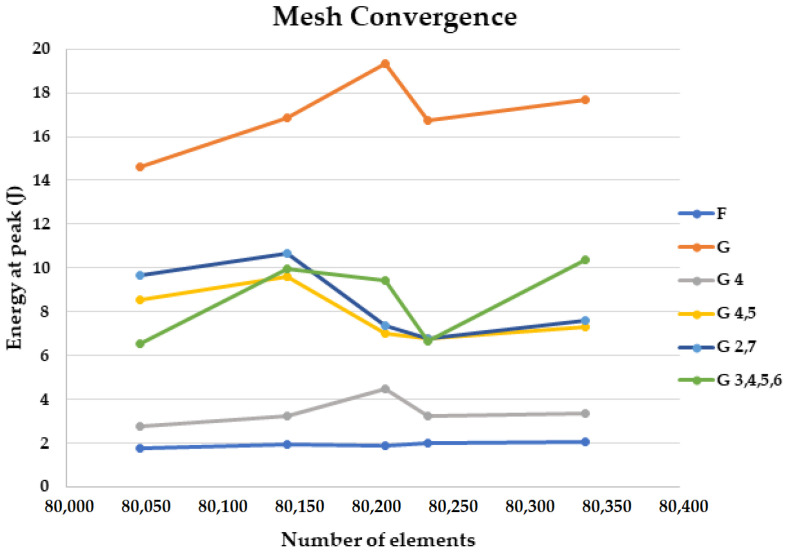
Convergence analysis for energy at peak (J).

In the Model section of the LS-DYNA module, the various layers were assigned the appropriate fabric materials along with defining the velocity of the impactor and various other parameters. The meshing was done using the multi zone, edge sizing and automatic method. Frictional interaction was defined between the layers. The convergence analysis was carried out, the mesh was refined and the simulations were conducted [76,77,78]. From the results obtained through convergence analysis, as shown in Figure 7, it was found that the number of elements could be varied from a minimum of 80,048 to a maximum of 80,339 and the energy at peak for all the samples showed a slight increase with a finer mesh and a slight decrease with a coarser mesh. It was found that the optimum number of elements to run the simulations would be 80,208 with 91,882 nodes and a mesh size of 3.54 mm.

Rigid body constraints were utilized for the impactor so as to keep the impactor steady.

## 3. Results & Discussion

### 3.1. Experimental Results

A total of 18 samples were tested for their low velocity drop-weight impact characteristics according to the ASTM D 7136 [74]. There were three repetitions of each sample tested, and values given in Table 3 are the averages of those results. The variations were minimum and within 5–7% CV.

Test Conditions:

Drop Height (mm): 942.436
Total Mass (kg): 5.410
Impact Energy (J): 50
Velocity at Start (m/s): 4.25

**Figure 8:**
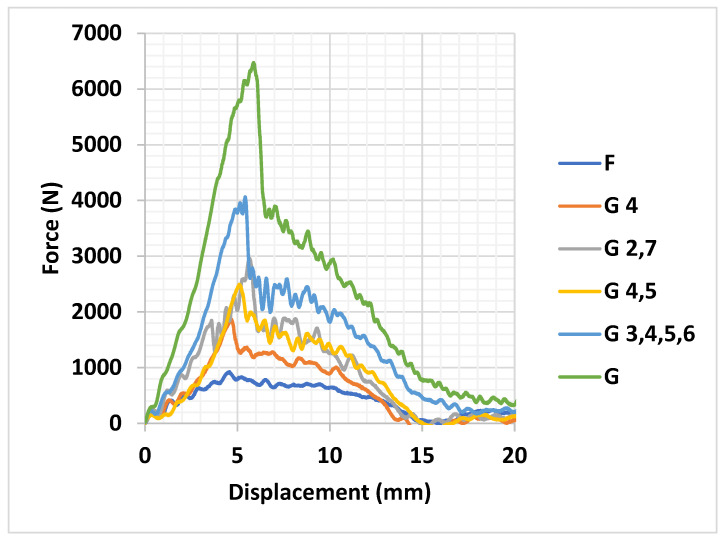
Force *v*/*s* displacement curves for composite samples.

The force *v*/*s* displacement curves in Figure 8 for the pure composite samples and the hybrid composite samples indicate the maximum (peak) force along with the maximum (peak) deformation experienced by the samples when subjected to an impact.

**Figure 9:**
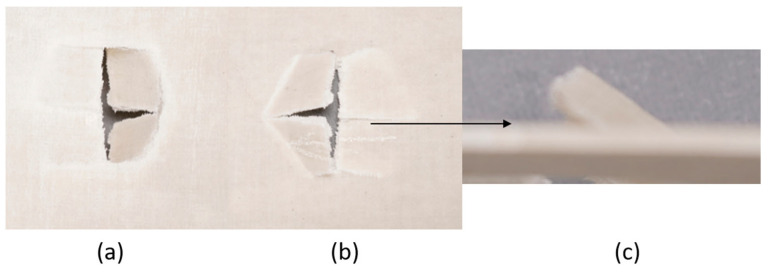
(**a**) Face; (**b**) back; (**c**) protrusion on back of sample 1: F due to impact.

**Figure 10:**
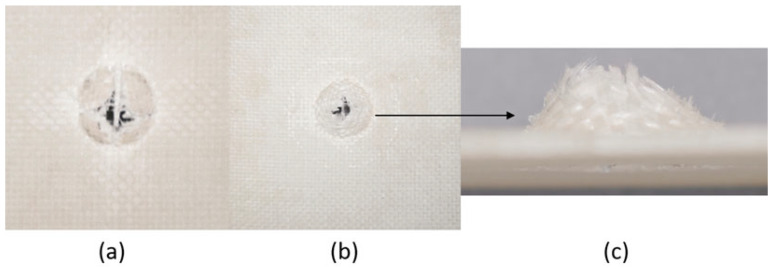
(**a**) Face; (**b**) back; (**c**) protrusion on back of sample 2: G due to impact.

**Figure 11:**
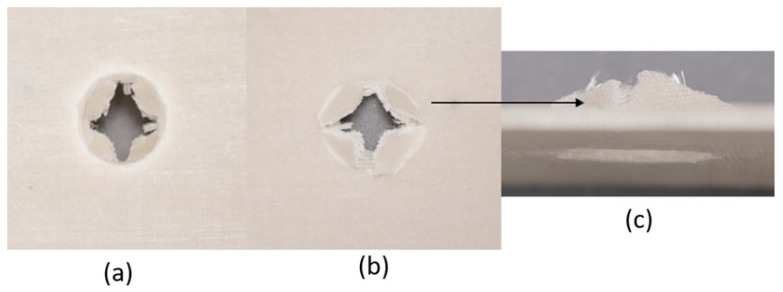
(**a**) Face; (**b**) back; (**c**) protrusion on back of sample 3: G 4 due to impact.

**Figure 12:**
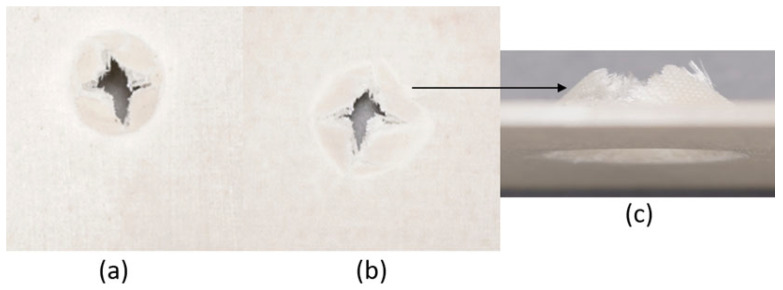
(**a**) Face; (**b**) back; (**c**) protrusion on back of sample 4: G 4, 5 due to impact.

**Figure 13:**
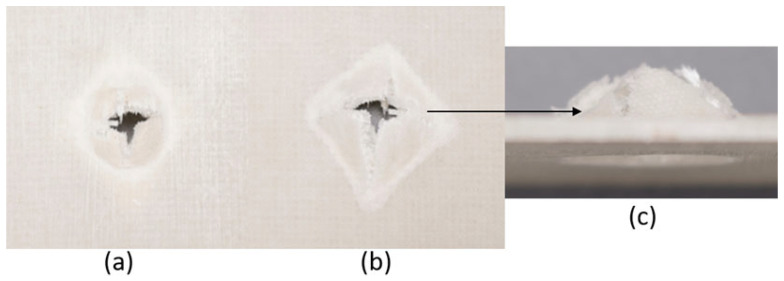
(**a**) Face; (**b**) back; (**c**) protrusion on back of sample 5: G 2, 7 due to impact.

**Figure 14:**
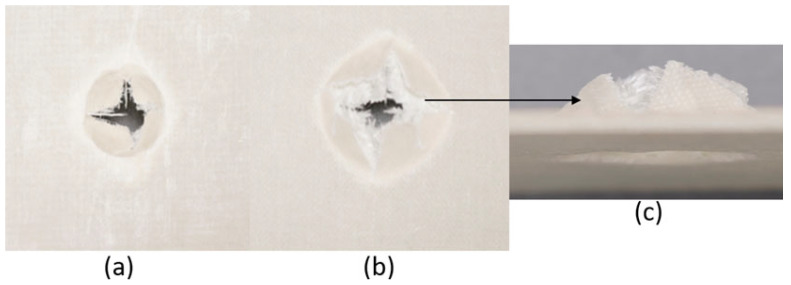
(**a**) Face; (**b**) back; (**c**) protrusion on back of sample 6: G 3, 4, 5, 6 due to impact.

The series of images shown in Figure 9, Figure 10, Figure 11, Figure 12, Figure 13 and Figure 14, show the face and back of the sample after impact.

Observing the results of the tests, it was seen that the pure flax composite laminates had the least force at peak when compared with the other pure composite laminate of glass and with the other hybrid composite laminates. While comparing the pure glass composite laminate with the other hybrid composite laminates, it can be observed that the force at peak was the highest for the pure glass composite laminate. Among the hybrid composite laminates, the composite with four layers of glass fabric had the highest force at peak and the composite laminates with two layers of glass fabric were almost similar, while the composite laminate with only one layer of glass fabric exhibited the least force at peak.

The samples were also tested by lining the composite laminates with a layer of EPS (expanded polystyrene) foam which had a density of 50 kg/mm^3^; the EPS foam applied had a thickness of 10 mm. Table 4 gives the results of the drop-weight impact tests conducted on the samples lined with EPS foam.

**Figure 15:**
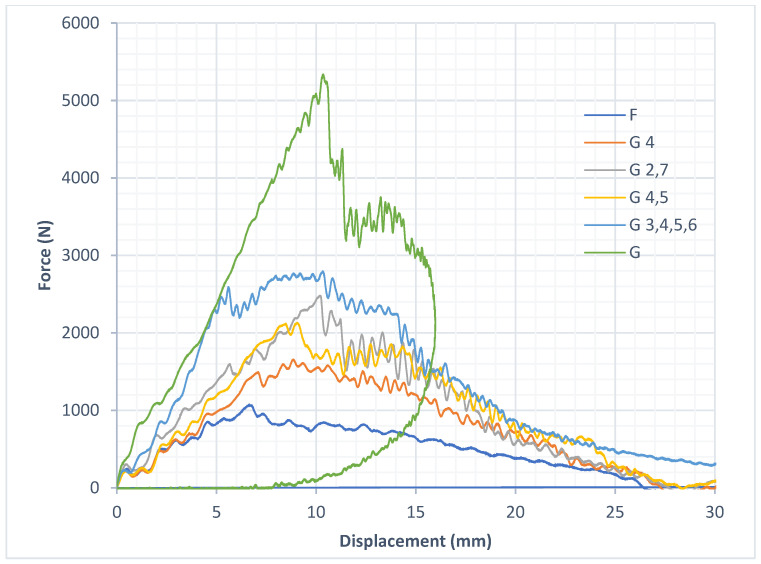
Force *v*/*s* displacement curves for samples lined with EPS foam.

Figure 15 shows the force *v*/*s* deformation curves of the samples lined with EPS foam.

In Figure 15 we can observe that the Sample G shows a peculiar curve where a closed loop was generated. This can be attributed to the rebounding of the impactor. This rebounding of the impactor represents the transfer of energy, first from the impactor to the composite sample, and then from the composite sample to the impactor.

**Figure 16:**
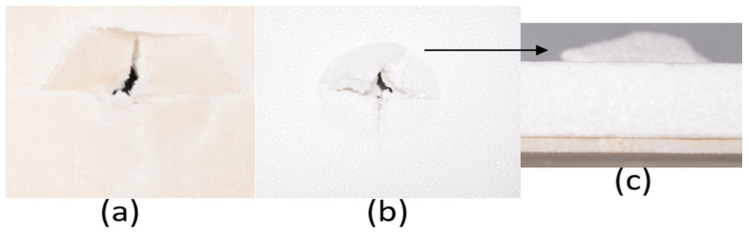
(**a**) Face and (**b**) back of sample 1: F lined with EPS foam; (**c**) protrusion of EPS foam due to impact on sample 1: F.

**Figure 17:**
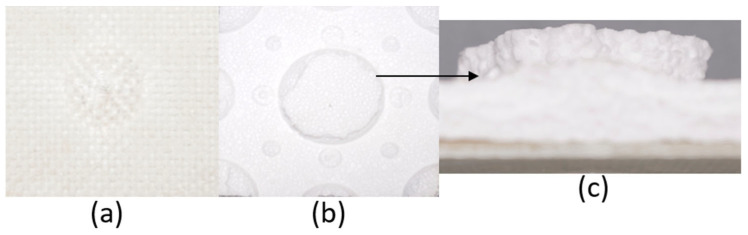
(**a**) Face and (**b**) back of sample 2: G lined with EPS foam; (**c**) protrusion of EPS foam due to impact on sample 2: G.

**Figure 18:**
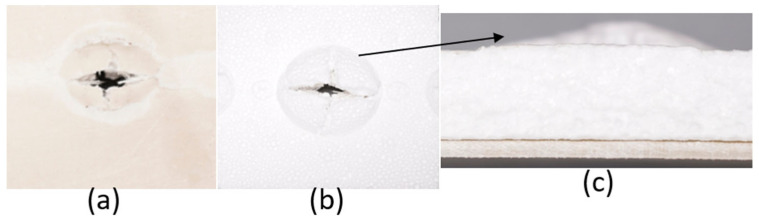
(**a**) Face and (**b**) back of sample 3: G 4 lined with EPS foam; (**c**) protrusion of EPS foam due to impact on sample 3: G 4.

**Figure 19:**
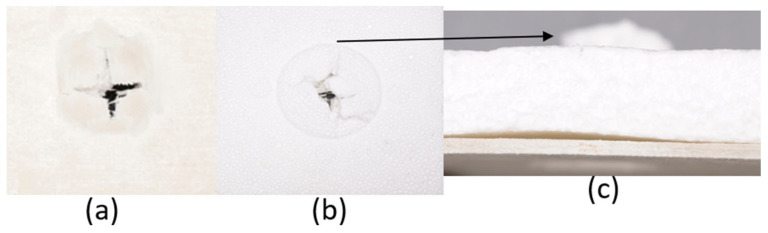
(**a**) Face and (**b**) back of sample 4: G 4, 5 lined with EPS foam; (**c**) protrusion of EPS foam due to impact on sample 4: G 4, 5.

**Figure 20:**
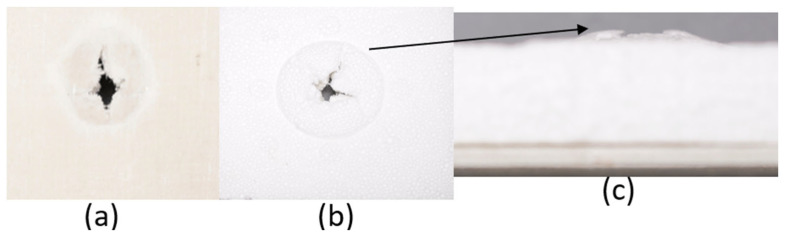
(**a**) Face and (**b**) back of sample 5: G 2, 7 lined with EPS foam; (**c**) protrusion of EPS foam due to impact on sample 5: G 2, 7.

**Figure 21:**
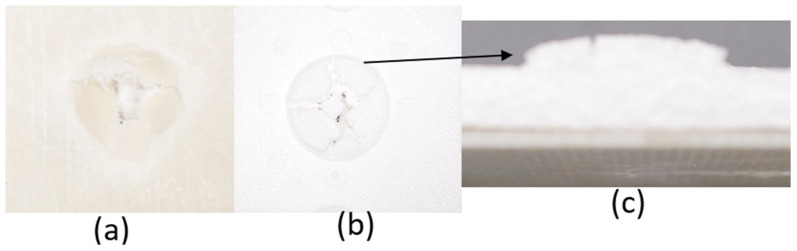
(**a**) Face and (**b**) back of sample 6: G 3, 4, 5, 6 lined with EPS foam; (**c**) protrusion of EPS foam due to impact on sample 6: G 3, 4, 5, 6.

The series of images shown in Figure 16, Figure 17, Figure 18, Figure 19, Figure 20 and Figure 21 show the face and back of the samples lined with EPS foam after impact.

Observing the results of the tests, it was seen that the pure flax composite laminates had the least force at peak when compared with the other pure composite laminate of glass and with the other hybrid composite laminates. While comparing the pure glass composite laminate with the other hybrid composite laminates, it can be observed that the force at peak was the highest for the pure glass composite laminate. Among the hybrid composite laminates, the composite with four layers of glass fabric had the highest force at peak and the composite laminates with two layers of glass fabric were almost similar, while the composite laminate with only one layer of glass fabric exhibited the least force at peak. Although the observation here was similar to the observation made from the tests conducted on the composite laminates not lined with EPS foam, it can be said that the presence of the foam for the set of these composite laminates affected the energy at peak values. The addition of foam reduces the contact stiffness, and thus upon impact the instantaneous peak force is lower as the foam gets crushed and dissipates the energy. When the values of energy at peak are compared for the laminates lined with EPS foam and laminates not lined with EPS foam, it can be seen that the values of energy at peak for each laminate is higher with EPS foam.

### 3.2. Simulation of Samples (Not Lined with EPS Foam)

**Figure 22:**
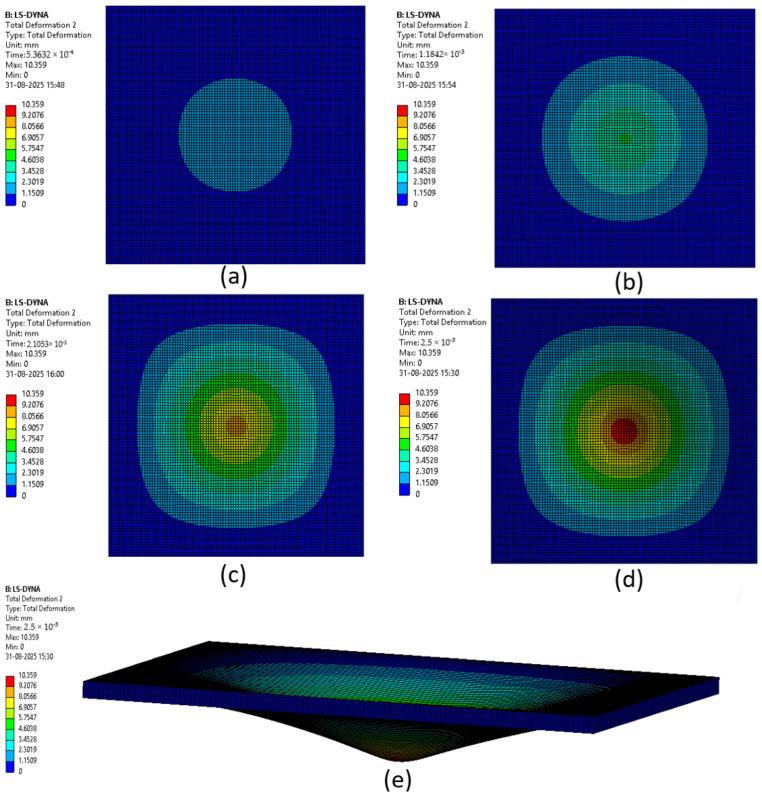
Phases in simulation of Sample 1: F. (**a**) Composite as impactor makes contact with surface initially; (**b**) initiation of impact; (**c**) progression of force being applied, leading to deformation of sample; (**d**) final stage where peak force was applied on composite; (**e**) deformation of composite due to peak force applied.

**Figure 23:**
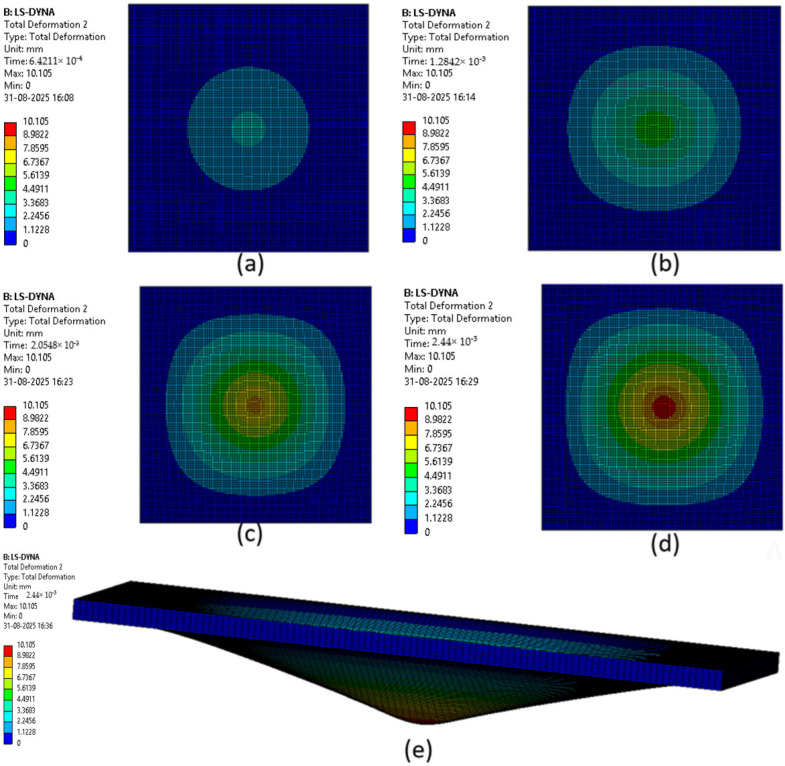
Phases in simulation of Sample 2: G. (**a**) Composite as impactor makes contact with surface initially; (**b**) initiation of impact; (**c**) progression of force being applied leading to deformation of sample; (**d**) final stage where peak force was applied on composite; (**e**) deformation of composite due to peak force applied.

**Figure 24:**
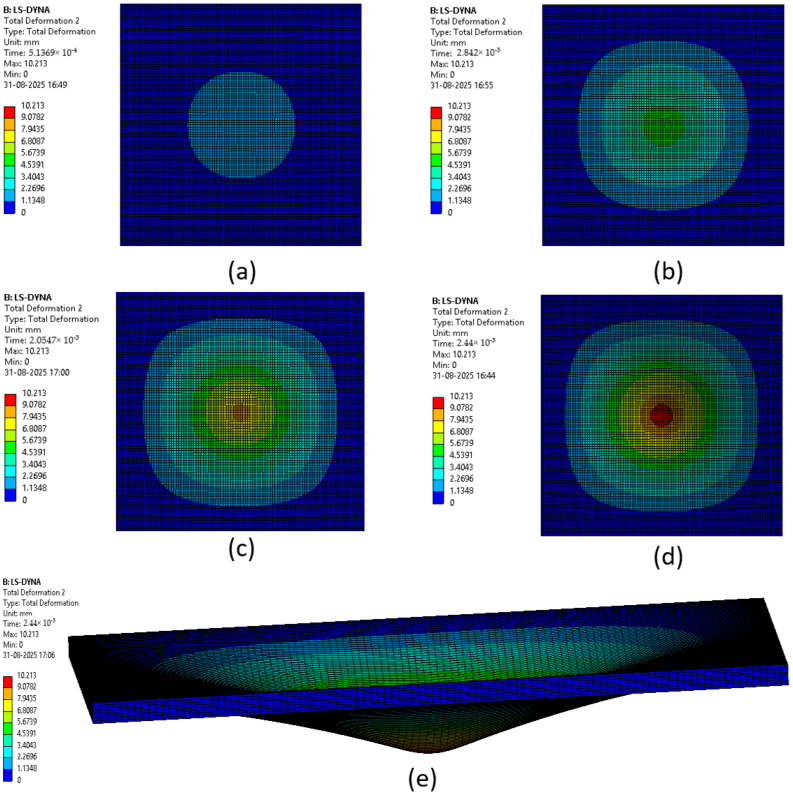
Phases in simulation of Sample 3: G 4. (**a**) Composite as impactor makes contact with surface initially; (**b**) initiation of impact; (**c**) progression of force being applied leading to deformation of sample; (**d**) final stage where peak force was applied on composite; (**e**) deformation of composite due to peak force applied.

**Figure 25:**
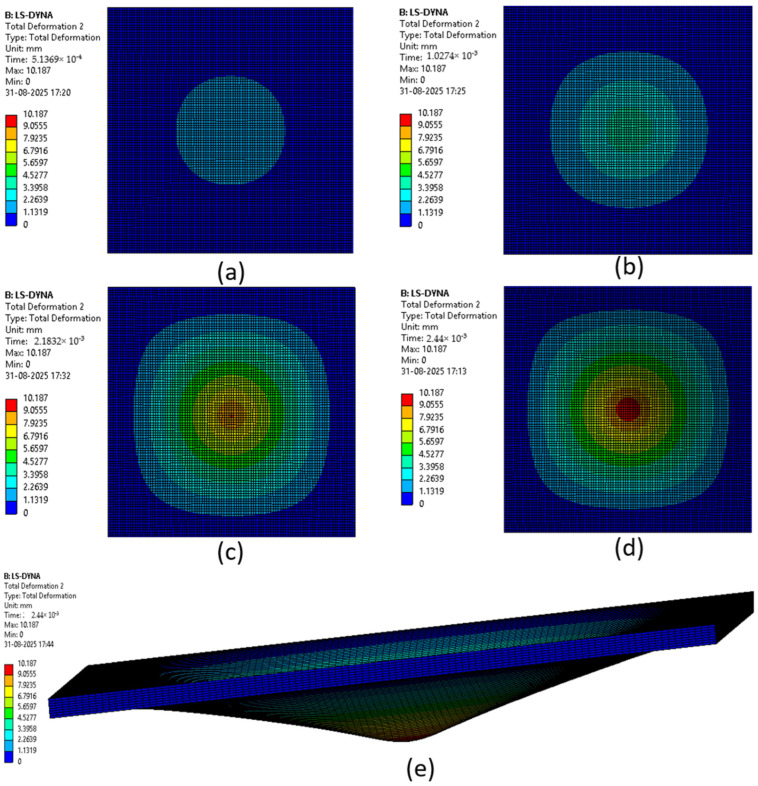
Phases in simulation of Sample 4: G 4, 5. (**a**) Composite as impactor makes contact with surface initially; (**b**) initiation of impact; (**c**) progression of force being applied leading to deformation of sample; (**d**) final stage where peak force was applied on composite; (**e**) deformation of composite due to peak force applied.

**Figure 26:**
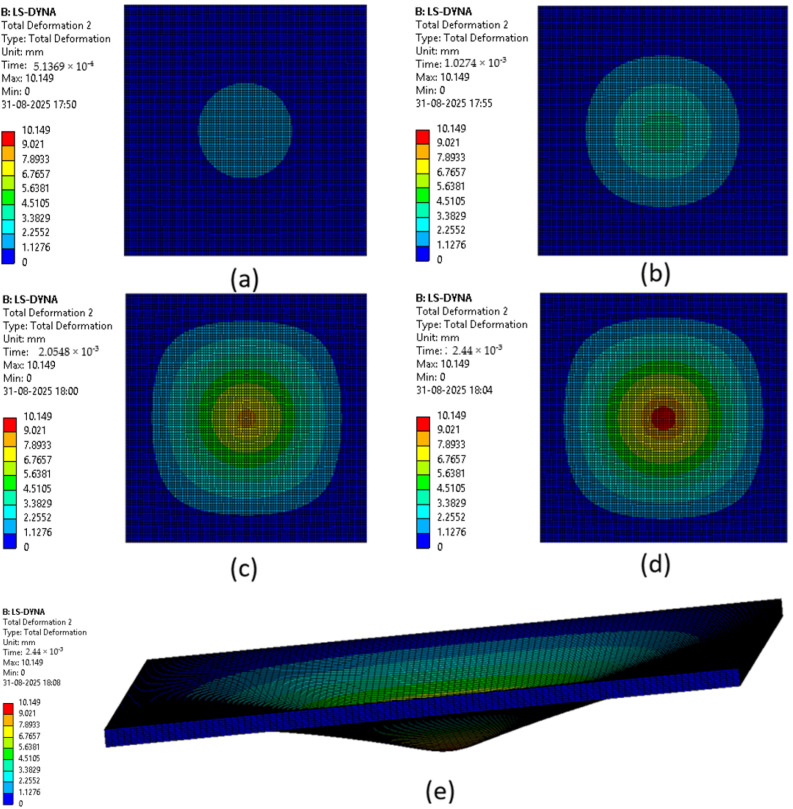
Phases in simulation of Sample 5: G 2, 7. (**a**) Composite as impactor makes contact with surface initially; (**b**) initiation of impact; (**c**) progression of force being applied leading to deformation of sample; (**d**) final stage where peak force was applied on composite; (**e**) deformation of composite due to peak force applied.

**Figure 27:**
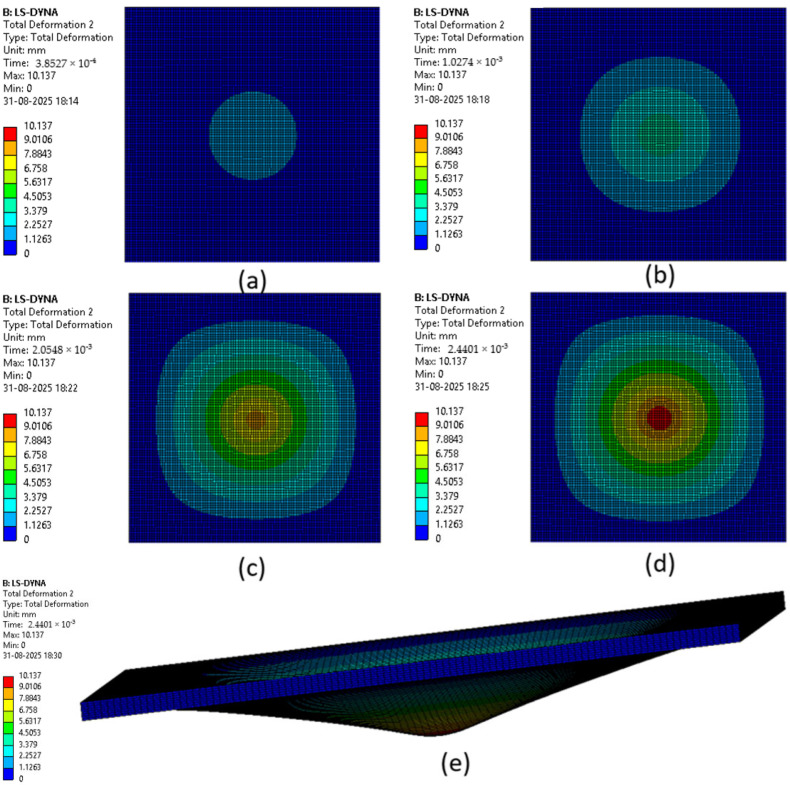
Phases in simulation of Sample 6: G 3, 4, 5, 6. (**a**) Composite as impactor makes contact with the surface initially; (**b**) initiation of impact; (**c**) progression of force being applied leading to deformation of sample; (**d**) final stage where peak force was applied on composite; (**e**) deformation of composite due to peak force applied.

The various phases which a composite laminate goes through while being simulated are represented in Figure 22, Figure 23, Figure 24, Figure 25, Figure 26 and Figure 27. Along with these phases, the figures also show the maximum deformation endured by the sample before breaking. This deformation is known as the peak deformation or deformation at peak and the force applied here is the peak force.

Figure 22 represents a series of images giving the phases of testing Sample 1: F; it can be seen from the images that initially the deformation is low, while in the final stages it can be observed that the deformation of the sample is high. This deformation leads to breaking of the composite laminate. The maximum deformation experienced by the laminate before breaking is also represented below the series of the images representing the phases.

Figure 23 represents a series of images giving the phases of testing Sample 2: G; it can be seen from the images that initially the deformation is low, while in the final stages it can be observed that the deformation of the sample is high. This deformation leads to breaking of the composite laminate. The maximum deformation experienced by the laminate before breaking is also represented below the series of the images representing the phases.

Figure 24 represents a series of images giving the phases of testing Sample 3: G 4; it can be seen from the images that initially the deformation is low, while in the final stages it can be observed that the deformation of the sample is high. This deformation leads to breaking of the composite laminate. The maximum deformation experienced by the laminate before breaking is also represented below the series of the images representing the phases.

Figure 25 represents a series of images giving the phases of testing Sample 4: G 4, 5; it can be seen from the images that initially the deformation is low, while in the final stages it can be observed that the deformation of the sample is high. This deformation leads to breaking of the composite laminate. The maximum deformation experienced by the laminate before breaking is also represented below the series of the images representing the phases.

Figure 26 represents a series of images giving the phases of testing Sample 5: G 2, 7; it can be seen from the images that initially the deformation is low, while in the final stages it can be observed that the deformation of the sample is high. This deformation leads to breaking of the composite laminate. The maximum deformation experienced by the laminate before breaking is also represented below the series of the images representing the phases.

Figure 27 represents a series of images giving the phases of testing Sample 6: G 3, 4, 5, 6; it can be seen from the images that initially the deformation is low, while in the final stages it can be observed that the deformation of the sample is high. This deformation leads to breaking of the composite laminate. The maximum deformation experienced by the laminate before breaking is also represented below the series of the images representing the phases.

The simulations performed were in agreement with the results from testing the samples and it was seen that Sample 1: F exhibits maximum deformation at the lowest force and Sample 2: G exhibits maximum deformation at the highest force. The following Table 5 shows a comparison between the maximum (peak deformation) deformation and maximum (peak force) obtained when tested and simulated.

From Table 5, we can conclude that the error between the tested results and simulated results for displacement at peak varies from a minimum of 3.32% to a maximum of 10.73%, the force at peak varies from a minimum of 0.06% to a maximum of 17.14%, the energy at peak—which is the energy absorbed by the sample at the point when the impact force reaches the maximum—varies from a minimum of 3.89% to a maximum of 61.73% and the energy at puncture—which is the total energy absorbed by the sample until puncture—varies from a minimum of 0.007% to a maximum of 0.066%.

The point of failure of the sample occurred at a very small time period as can be seen in the Figure 21, Figure 22, Figure 23, Figure 24 and Figure 25 where the time can be seen as (2.5 × 10^−3^ s or 2.44 × 10^−3^ s). Hence, the data generated up to this time was very little and could not be matched to the amount of data gathered when the test was conducted physically, which is why the graphs for deformations were not created.

The variation between the tested and simulated results can be attributed to the fact that discrepancies could have crept in while producing the samples. Table 6 shows the results obtained after testing the lined and not lined composite laminates.

From Table 7, we can see that the energy at peak for the laminates lined with EPS foam has increased drastically; this in turn would mean that the energy absorbed by the laminates lined with EPS foam is higher. The energy at puncture and the percentage by which it increased for the set of samples lined with EPS foam is given in Table 7 and Table 8 shows the percentage increase in the displacement at peak when comparing the two sets of composite laminates. The increase in displacement also plays into cementing the fact that the energy absorbed is higher.

## 4. Conclusions

Numerous studies have been performed on the effect of low impact on composites made from glass or carbon fiber. In this study, pure laminates made from glass and flax fabrics, along with hybrid composites made using a combination of flax fabrics and glass fabrics, lined with EPS foam and not lined with EPS foam were tested at room temperature. The results obtained by testing the composite laminates not lined with EPS foam were compared with simulated results obtained from simulating the composite laminates not lined with EPS foam.

Upon comparing the results of laminates lined with EPS foam and laminates not lined with EPS foam, it can be seen clearly that the energy at peak for the laminates lined with EPS foam is higher. This is because the energy applied to the laminate is transferred through the various layers of the composite and then to the layer of foam, and the foam absorbs the energy applied to a certain degree. From Table 7, it can be clearly seen that by applying a lining of foam to the laminate which absorbed the least amount of energy (Sample1: F), the amount of energy absorbed by the laminate has increased by a percentage of 82.95. When we shine a light onto the percentage increase in the displacement at peak, it can be observed that the displacements at peak endured by the composite laminates lined with EPS foam are significantly higher than displacements at peak for the composite laminates not lined with EPS foam. This increase in displacement at peak also lends a hand in corroborating that applying a lining of EPS foam on the composite laminates helps in increasing the energy absorbed at peak. This is again reinforced when we examine the energy at puncture values for both the laminate sets. A clear increase in the energy at puncture values for the laminates lined with EPS foam is shown when compared with the values obtained for the laminates not lined with EPS foam. When we compare the values obtained in Table 3 and Table 4, we see that the samples containing glass fibers show a decrease in the peak force; this can be attributed to the fact that foam can reduce the contact stiffness, and upon impact the instantaneous peak force is lower as the foam gets crushed and dissipates the energy leading to higher absorption of energy up to the point of puncture. Thus, it can be said that by applying a lining of foam on the composite laminates, the amount of energy absorbed by the laminates can be increased, which would prove to be beneficial when used as a structural component.

All the results obtained for the composite laminates not lined with EPS foam and laminates lined with EPS foam from experimental test and simulations pointed towards one observation, that the energy at peak, displacement at peak, force at peak and energy at puncture increased as the number of layers of glass fabric increased. This increase can be attributed to the fact that the strength of the glass fiber is inherently greater than that of the flax fiber. Thus, it can be concluded that the increase in the number of glass fabric layers leads to improved mechanical performance of the composite laminates. Apart from this, we can also observe that the effect of the stacking sequence also plays a role. It is evident from the results obtained that the samples which have two layers of glass fabrics achieve almost similar results to the sample containing four layers of glass. The effect of the stacking of glass fabrics in different positions can be studied further. This also shows that if we study the effect of the stacking, we can achieve the desired levels of mechanical properties while utilizing lower number of glass layers, thus playing into the fact that we are trying to make the composites more environmentally friendly.

Since the multilayered samples used in this research were specially designed with a combination of eight layers of flax and glass fabrics in different stacking sequences, no comparable data could be found from existing publications. When the results obtained via simulations were compared with the tested results, it was be seen that the error in deformation varies between 3.32% to 10.73% and the error in the force at peak varies from 0.06% to 17.14%. This variation in the values between the tested and simulated samples may be a result of boundary condition and loading configuration applied during simulations, which could affect the distribution of force and the contact time. On the other hand, the of presence of voids in the composite laminates, the variation of the diameter of the natural fibers used, and their morphology may also contribute to the variations in the results which have been observed.

It is necessary to consider the design aspects (e.g., dimension, shape, curvature, geometry, location and joining elements) while designing such composites in specific machine elements in automotives (e.g., bumper, spring, battery box, storage cabinet or other interior components).

## Figures and Tables

**Table 1:** Material properties. Adapted from [1].

Properties	Flax	Glass
Density	1.5 ± 0.1 (g/cm^3^)	2.48 ± 0.2 (g/cm^3^)
Fiber Diameter	20 ± 1.2 (µm)	21 ± 1.1 (µm)
GSM of Fabric	600 ± 10 (g/m^2^)	600 ± 25 (g/m^2^)
Tensile Strength	2.47 ± 0.05 (GPa)	4.65 ± 0.15 (GPa)

**Table 2:** Samples prepared. Adapted from [2].

Samples	Layers	Orientation (°)
F	FFFFFFFF	0/90
G	GGGGGGGG	0/90
G 4	FFFGFFFF	0/90
G 4, 5	FFFGGFFF	0/90
G 2, 7	FGFFFFGF	0/90
G 3, 4, 5, 6	FFGGGGFF	0/90

**Table 3:** Results of low velocity drop-weight impact tests.

Samples	F_peak_-NL (N)	D_peak_-NL (mm)	E_peak_-NL (J)	E_pun_-NL (J)
F	935.249	4.499	2.122	7.208
G	6504.317	5.685	16.905	25.597
G 4	1950.827	4.774	3.558	6.563
G 4, 5	2535.098	5.127	4.322	12.274
G 2, 7	2977.013	5.656	7.103	7.982
G 3, 4, 5, 6	4098.303	5.280	9.024	12.515

F_peak_-NL—force at peak—not lined with EPS foam; D_peak_-NL—displacement at peak—not lined with EPS foam; E_peak_-NL—energy at peak—not lined with EPS foam; E_pun_-NL—energy at puncture—not lined with EPS foam.

**Table 4:** Results of EPS foam lined samples.

Samples	F_peak_-L (N)	D_peak_-L (mm)	E_peak_-L (J)	E_pun_-L (J)
F	1114.51	6.688	3.882	9.38
G	5727.212	11.802	33.532	34.565
G 4	1746.588	9.855	9.215	14.825
G 4, 5	2150.24	9.608	12.575	20.6053
G 2, 7	2711.781	11.501	16.054	18.659
G 3, 4, 5, 6	3870.022	9.891	18.694	26.33

F_peak_-L—force at peak—lined with EPS foam; D_peak_-L—displacement at peak—lined with EPS foam; E_peak_-L—energy at peak—lined with EPS foam; E_pun_-L—energy at puncture—lined with EPS foam.

**Table 5:** Comparison between tested and simulated samples.

	Tested Results	Simulated Results	Error %
Sample 1: F			
D_peak_-NL (mm)	4.49	4.1125	8.4%
F_peak_-NL (N)	935.241	935.87	0.06%
E_peak_-NL (J)	2.122	1.92	9.51%
E_pun_-NL (J)	7.208	7.204	0.055%
Sample 2: G			
D_peak_-NL (mm)	5.685	5.4962	3.32%
F_peak_-NL (N)	6504.317	6484	0.31%
E_peak_-NL (J)	16.905	19.34	14.4%
E_pun_-NL (J)	25.597	25.58	0.066%
Sample 3: G 4			
D_peak_-NL (mm)	4.774	4.576	4.14%
F_peak_-NL (N)	1950.827	1948.8	0.10%
E_peak_-NL (J)	3.558	4.46	25.35%
E_pun_-NL (J)	6.563	6.564	0.015%
Sample 4: G 4, 5			
D_peak_-NL (mm)	5.127	5.5852	8.93%
F_peak_-NL (N)	2535	2502.8	1.27%
E_peak_-NL (J)	4.322	6.99	61.73%
E_pun_-NL (J)	12.274	12.27	0.032%
Sample 5: G 2, 7			
D_peak_-NL (mm)	5.656	5.049	10.73%
F_peak_-NL (N)	2977.613	2923.3	1.82%
E_peak_-NL (J)	7.103	7.38	3.89%
E_pun_-NL (J)	7.982	7.98	0.025%
Sample 6: G 3, 4, 5, 6			
D_peak_-NL (mm)	5.280	5.540	4.92%
F_peak_-NL (N)	4098.303	3395.7	17.14%
E_peak_-NL (J)	9.024	9.41	4.27%
E_pun_-NL (J)	12.515	12.516	0.007%

Force at peak—not lined with EPS foam—F_peak_-NL; Displacement at peak—not lined with EPS foam—D_peak_-NL; Energy at peak—not lined with EPS foam—E_peak_-NL; Energy at puncture—not lined with EPS foam—E_pun_-NL.

**Table 6:** Comparing results obtained after testing lined and not lined composite laminates.

Samples	F_peak_-NL (N)	D_peak_-NL (mm)	E_peak_-NL (J)	E_pun_-NL (J)	F_peak_-L (N)	D_peak_-L (mm)	E_peak_-L (J)	E_pun_-L (J)
F	935.249	4.499	2.122	7.208	1114.51	6.688	3.882	9.38
G	6504.317	5.685	16.905	25.597	5727.212	11.802	33.532	34.565
G 4	1950.827	4.774	3.558	6.563	1746.588	9.855	9.215	14.825
G 4, 5	2535.098	5.127	4.322	12.274	2407.971	10.608	12.575	20.6053
G 2, 7	2977.013	5.656	7.103	7.982	2711.781	11.501	16.054	18.659
G 3, 4, 5, 6	4098.303	5.280	9.024	12.515	3870.022	9.891	18.694	26.33

**Table 7:** Percentage increase in energy at peak for laminates lined with EPS foam.

Samples	E_peak_-NL (J)	E_peak_-L (J)	Increase (%)	E_pun_-NL (J)	E_pun_-L (J)	Increase (%)
F	2.122	3.882	82.95	7.208	9.38	30.13
G	16.905	33.532	98.35	25.597	34.565	35.03
G 4	3.558	9.215	158.99	6.563	14.825	125.9
G 4, 5	4.322	12.575	190.95	12.274	20.6053	67.89
G 2, 7	7.103	16.054	126.01	7.982	18.659	133.7
G 3, 4, 5, 6	9.024	18.694	107.15	12.515	26.33	110.3

**Table 8:** Percentage increase in displacement at peak for laminates lined with EPS foam.

Samples	D_peak_-NL	D_peak_-L	Percentage Increase (%)
F	4.499	6.688	48.65
G	5.685	11.802	107.59
G 4	4.774	9.855	106.43
G 4, 5	5.127	10.608	106.90
G 2, 7	5.656	11.501	103.34
G 3, 4, 5, 6	5.280	9.891	87.32

## Data Availability

The original contributions presented in the study are included in the article. Further inquiries can be directed to the corresponding author.
